# Randomised-crossover clinical trial on the substantivity of a single application of a gel containing chlorhexidine and o-cymen-5-ol on the oral biofilm and saliva

**DOI:** 10.1186/s12903-024-05042-7

**Published:** 2024-10-19

**Authors:** B. Suárez-Rodríguez, A. Regueira-Iglesias, T. Blanco-Pintos, A. Sánchez-Barco, N. Vila-Blanco, C. Balsa-Castro, M. J. Carreira, I. Tomás

**Affiliations:** 1 Oral Sciences Research Group, Special Needs Unit, Department of Surgery and Medical- Surgical Specialties, School of Medicine and Dentistry, Universidade de Santiago de Compostela, Fundación Instituto de Investigación Sanitaria de Santiago (FIDIS), Santiago de Compostela, A Coruña Spain; 2 Centro Singular de Investigación en Tecnoloxías Intelixentes (CiTIUS) and Departamento de Electrónica e Computación, Universidade de Santiago de Compostela, Fundación Instituto de Investigación Sanitaria de Santiago (FIDIS), Santiago de Compostela, A Coruña Spain

**Keywords:** Chlorhexidine, Cymenol, Gel, Saliva, Oral biofilm, Confocal laser scanning microscopy, Bacterial viability, Automated computation

## Abstract

**Background:**

No clinical trials have evaluated the antimicrobial activity and substantivity of gel formulations containing chlorhexidine (CHX) and cymenol.

**Objective:**

To compare the in situ antimicrobial effect and substantivity of a new 0.20% CHX + cymenol gel (test) with the current 0.20% CHX gel formulation (control) on salivary flora and dental plaque biofilm up to seven hours after a single application.

**Methods:**

A randomised-crossover clinical trial was conducted with 29 orally healthy volunteers participating in the development of Experiments 1 (saliva) and 2 (dental plaque biofilm). All subjects participated in both experiments and were randomly assigned to receive either the test or control gels. Samples were collected at baseline and five minutes and one, three, five, and seven hours after a single application of the products. The specimens were processed using confocal laser scanning microscopy after staining with the LIVE/DEAD^®^ BacLight™ solution. Bacterial viability (BV) was quantified in the saliva and biofilm samples. The BV was calculated using the DenTiUS Biofilm software.

**Results:**

In Experiment 1, the mean baseline BV was significantly reduced five minutes after application in the test group (87.00% *vs.* 26.50%; *p* < 0.01). This effect was maintained throughout all sampling times and continued up to seven hours (40.40%, *p* < 0.01). The CHX control followed the same pattern. In Experiment 2, the mean baseline BV was also significantly lower five minutes after applying the test gel for: (1) the total thickness of biofilm (91.00% *vs*. 5.80%; *p* < 0.01); (2) the upper layer (91.29% *vs*. 3.94%; *p* < 0.01); and (3) the lower layer (86.29% *vs.* 3.83%; *p* < 0.01). The reduction of BV from baseline was observed for the full-thickness and by layers at all sampling moments and continued seven hours after application (21.30%, 24.13%, and 22.06%, respectively; *p* < 0.01). Again, the control group showed similar results. No significant differences between test and control gels were observed in either saliva or dental plaque biofilm at any sampling time.

**Conclusions:**

A 0.20% CHX + cymenol gel application demonstrates potent and immediate antimicrobial activity on salivary flora and *de novo* biofilm. This effect is maintained seven hours after application. Similar effects are obtained with a 0.20% CHX-only gel.

**Supplementary Information:**

The online version contains supplementary material available at 10.1186/s12903-024-05042-7.

## Introduction

The oral cavity harbours a complex microbiome whose bacteria are capable of organising into oral biofilms like dental plaque, causing conditions including dental caries and periodontitis [1, 2]. The removal of tooth plaque via brushing plays a crucial role in preventing these diseases [3, 4]. However, such mechanical methods are not always entirely effective because of factors including access to interproximal areas, the time spent brushing, or patient dexterity [5–7]. Consequently, coadjuvant methods based on chemical control have additional benefits for maintaining oral health [8–11].

Chlorhexidine gluconate (CHX) is a cationic bisbiguanide compound that is often used as a broad-spectrum oral antiseptic [12]. The mechanism of action of this active agent is based on the alteration or destruction of microbial cell membranes [12, 13]. Its widely documented efficacy makes CHX the gold-standard anti-plaque and anti-gingivitis agent [12, 14]. Essential oils (EO) are the other commonly used broad-spectrum antimicrobials [15]. These contain hundreds of chemical substances known for their anti-inflammatory or antioxidant properties [16, 17]. For its part, EOs act by altering the microbial cell wall and membrane permeability [18, 19].

A remarkable property of antiseptics such as those described above is substantivity, which is defined as residual antimicrobial activity in the mouth over time. This ability to adsorb to oral surfaces confers bacteriostatic activity, which results in a delayed regrowth of biofilm [20]. Substantivity can be affected by multiple factors, such as product-specific or subject-specific factors [21]. Among them, adhesion could lead to differences in the results of an antiseptic obtained with gel or rinse formulations [22].

In the EO family, o-cymen-5-ol (cymenol) is a poorly investigated, naturally phenolic compound derived from isopropyl cresol that helps maintain oral health [23–25]. As a high capacity to penetrate dental plaque biofilm [26–28] is attributed to EOs, combining cymenol with other active agents could enhance their effect and penetrability. A recent in vitro investigation has found higher bactericidal activity with a CHX + cymenol gel than with CHX-only and a higher degree of penetrability into the biofilm [29].

Epifluorescence and confocal laser scanning microscopy (CLSM) are among the tools employed in research to examine oral antiseptics’ substantivity in mouth samples. These microscopic techniques are complemented by the use of stains, such as the SYTO™ 9 and propidium iodide (PI), to visualise the structural arrangement of the bacteria and their viability [30]. Our group has extensive experience in the field of in situ studies of the antimicrobial effect of different antiseptics on salivary flora and biofilm using these methodologies [28, 31–37]. However, the traditional methodology employed for manually or semi-automatically quantifying microorganisms using microscopy images is a slow and tedious process that is also prone to error [38]. Software packages created to count bacteria automatically are much faster and produce more objective and reliable data [39–41]. One such program is the DenTiUS Biofilm tool developed by our research group, which can quantify bacterial viability (BV) and analyse its evolution over time [42].

Only one clinical trial to date has employed CLSM and an automated software package to analyse the in vivo substantivity of mouthwashes containing only cymenol or in combination with cetylpyridinium chloride (CPC) [43]. There have, however, been no in vivo clinical trials that have evaluated gel applications of cymenol combined with a more effective agent than CPC, e.g., CHX [44]. Consequently, the present study compares the in situ immediate and persistent antimicrobial effect (substantivity) of a gel containing 0.20% CHX and cymenol *vs.* the commonly used 0.20% CHX gel formulation (as a control). For this purpose, we used CLSM and DenTiUS Biofilm software on salivary flora and dental plaque biofilm samples taken from healthy individuals for up to seven hours after a single application.

## Materials and methods

This was a balanced, randomised, triple-blind, parallel, crossover study of the in situ substantivity of a 0.20% CHX + cymenol gel (Lacer^®^, Barcelona, Spain) *vs.* a 0.20% gel containing CHX alone (Lacer^®^, Barcelona, Spain). The project was approved by the Clinical Research Ethics Committee, Galicia, Spain (CEIC), where it is registered with the number 2021/478. The protocol for this trial and the CONSORT checklist are available as Additional files 1 and 2, respectively. The investigation was registered at ClinicalTrials.gov with the identification number NCT06437262 (date of registration 31/05/2024). The authors can confirm that all ongoing and related trials for this intervention are recorded and can be accessed via the following URL: https://clinicaltrials.gov/study/NCT06437262. All the procedures performed in the experiments were explained orally and in writing to each participant, and their written informed consent was obtained.

### Selection of the study group: inclusion and exclusion criteria

Potential participants were sought for voluntary enrolment in the trial at the setting of the Faculty of Medicine and Dentistry at the University of Santiago de Compostela (USC) in Spain between September 2022 and June 2023. All the volunteers were evaluated by two dentists who confirmed compliance with the established inclusion and exclusion criteria. The subjects included were systemically healthy adults aged 20–45 years with an excellent oral health status, which was defined as ≥ 24 permanent teeth with no evidence of gingivitis or periodontitis (bleeding on probing -BOP- <10%, probing pocket depth -PPD- ≤3 mm) [45] and no untreated caries at baseline. The exclusion criteria were as follows: smoker or former smoker; the presence of dental prostheses or orthodontic appliances; allergies to oral hygiene products; antibiotic treatment and routine use of oral antiseptics in the previous three months; and the presence of any systemic disease that could alter saliva production or composition. Lastly, as a withdrawal criterion, all the volunteers had to agree to participate in both of our experiments (explained below in detail); if they did not, they were excluded from the final analysis.

### Study experiments and gel application protocol

**Fig. 1:**
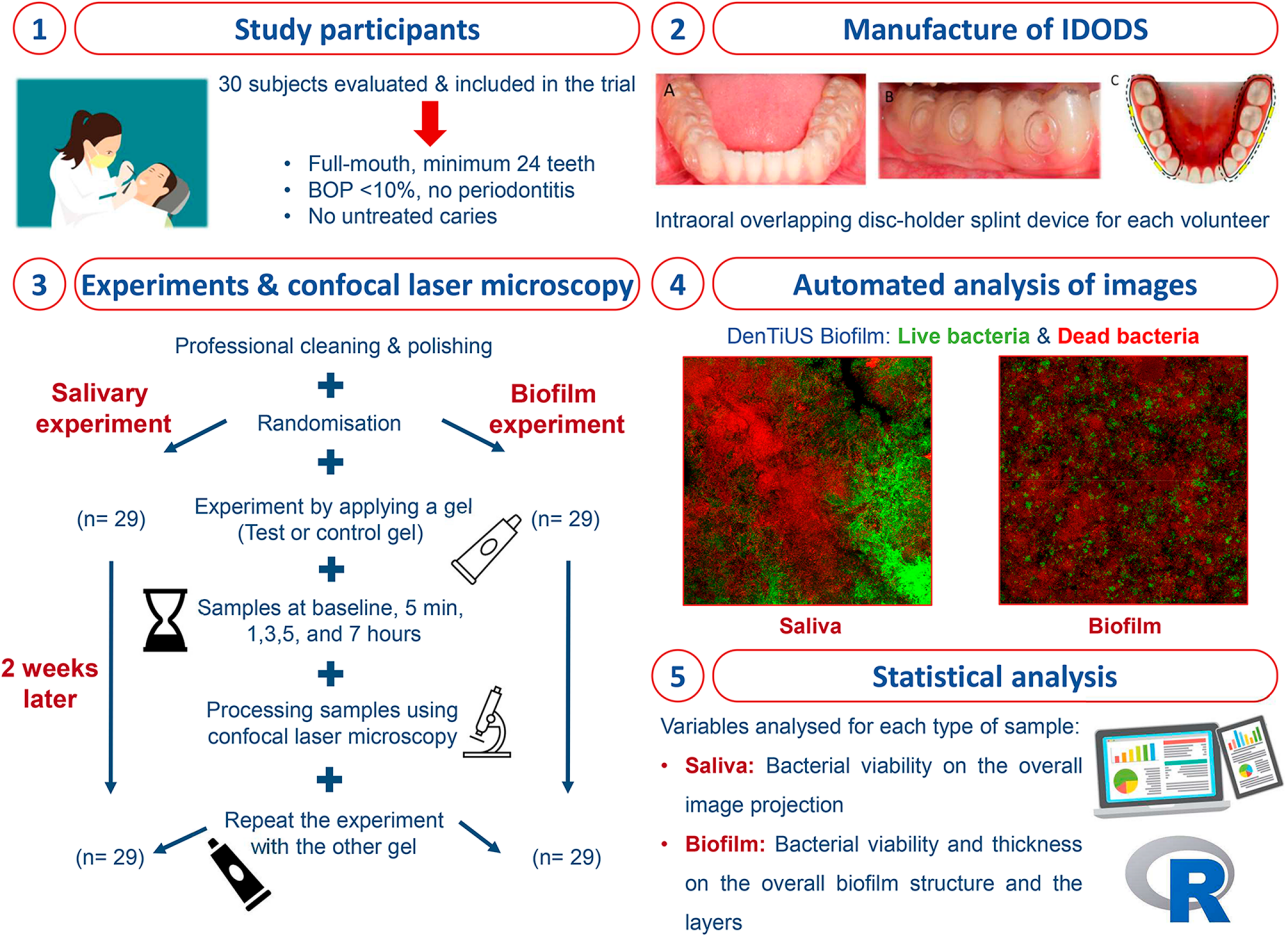
Flowchart of the development of this experiment

The application of the criteria above left 30 subjects for inclusion in the investigation. They all participated in developing the two experiments, the first involving salivary flora and the second dental plaque biofilm. Each experiment required two appointments. A single application of one of the gel formulations being evaluated was applied at each appointment for the particular experiment. The products tested were a 0.20% Lacer^®^ CHX gel with a new formulation containing cymenol (test gel) *vs.* a conventional 0.20% Lacer^®^ CHX gel without cymenol (control gel). Samples were collected at each appointment in baseline conditions (i.e., before application) and then at five minutes and one, three, five, and seven hours after application of the corresponding gel (Fig. 1).

Before starting the trial, all the volunteers underwent professional ultrasonic cleaning and rubber cup dental polishing. They were told not to brush their teeth or practice other oral hygiene measures between 48 h before the sampling day and when the last specimen was taken. At the sampling appointments, they were not allowed to eat or drink from 60 min before the collection of the first oral sample until after the last one had been obtained. This process started at 8.45 a.m. (baseline sample) and ended at 4.00 p.m. (sample obtained seven hours after gel application). Both the test and control gels were prepared in opaque tubes labelled with a participant number (from one to 30), and each one contained the amount required for a single application plus extra gel in the case of possible losses.

For the purpose of unbiased allocation, we implemented a balanced randomisation process using the R free-distribution software (version 4.4.0) [46]. This method generated a designation list, determining which gel each participant would receive during each experiment’s first and second appointments. All volunteers were subjected to both study gels in both experiments, with a minimum two-week rest period between them.

#### Experiment 1: salivary flora

The spitting method [47] was used to collect unstimulated saliva samples (1 ml) from each volunteer at the time points described above. The researcher applied the corresponding gel to the buccal and palatal/lingual tooth surfaces and gingival mucosa of both subjects’ mouth arches.

#### Experiment 2: dental plaque biofilm

For Experiment 2, a dental plaque biofilm study, an intraoral overlapping disc-holder splint device (IDODS, registered patent number ES 2380252 B2) was custom-made for each volunteer. This device, comprising two splints with six two-mm-diameter circular cavities each, was designed to encourage dental plaque biofilm growth on the glass discs. The device was positioned in the lower hemiarch, and the glass discs were exposed to the vestibular area but protected from cheek action by an external splint frame.

The volunteers wore the IDODS for 48 h to encourage dental plaque biofilm growth on the glass discs. They were instructed to only remove it from the oral cavity during meals (stored in an opaque container under humid conditions). After 48 h, the glass discs were removed one by one from the splints of each volunteer (from right to left, starting distally) at the time points described above. The investigator applied the corresponding gel to the entire exposed surface of the six discs ex vivo to ensure the integrity of the biofilm. After this application, the volunteers placed the IDODS back inside their mouths until the final sampling time of seven hours.

### Processing of the saliva and dental plaque biofilm samples using confocal laser microscopy

**Fig. 2:**
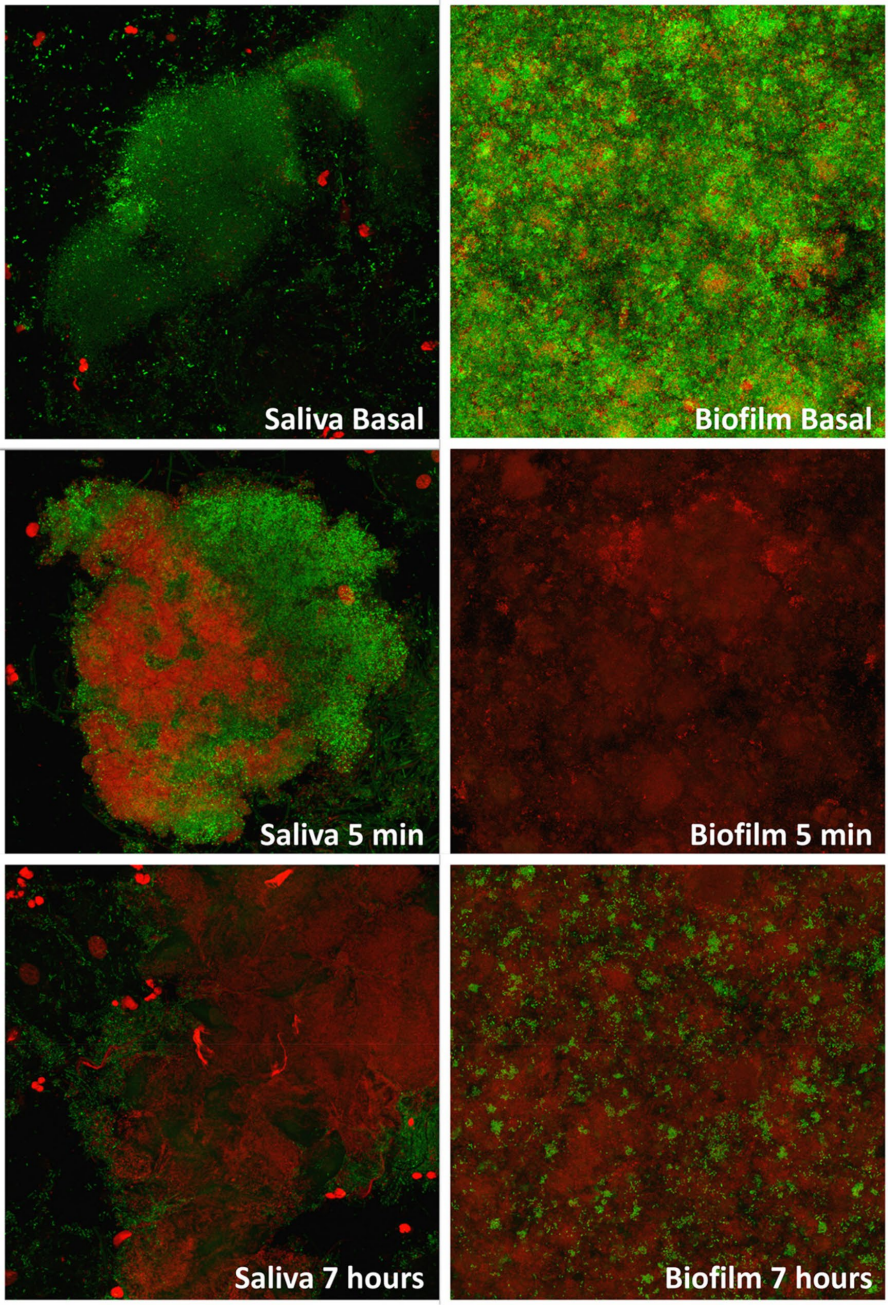
Images obtained by confocal microscopy and processed with DenTiUS Biofilm software after test gel application MIN: minutes

The LIVE/DEAD^®^ BacLight™ kit (Molecular Probes, Leiden, the Netherlands), consisting of SYTO™ 9 and PI, was used as a fluorescence solution to determine BV in the two experiments. The solution was prepared according to the manufacturer’s instructions, i.e., using 5 ml of sterile water, filtered with a 0.22 μm Millipore membrane filter (Millipore Ibérica S.A., Madrid, Spain) to achieve a 1:1 ratio of both fluorochromes and stored at -20 °C. SYTO™ 9 dye penetrates viable and non-viable cells due to its ability to penetrate intact membranes. However, PI penetrates only into damaged cells, as it only permeabilises cells with high membrane permeability. Both stain nucleic acids and can be monitored for their fluorescence, so double staining allows differentiation between them [48] (Fig. 2).

In Experiment 1, the saliva samples were centrifuged at 14,500 rpm for 10 min. The supernatant was discarded, and the pellet obtained was re-suspended in 100 µl of sterile water with 100 µl of the fluorescence solution. The bacterial suspension was shaken for homogenisation and stored in the dark at room temperature for 15 min.

In Experiment 2, the glass discs removed from the IDODS were immediately immersed in 150 µl of the LIVE/DEAD^®^ BacLight™ fluorescent solution and kept in a dark chamber at room temperature for 15 min.

The microscopic observations in both experiments were carried out by a researcher unaware of the study design. For this purpose, a Leica TCS SP5 X laser scanning spectral confocal microscope (Leica Microsystems Heidelberg GmbH, Mannheim, Germany) equipped with a white laser (WLL) (Research Infrastructures Area of University of Santiago de Compostela. Microscopy Unit, CIMUS) was employed.

### Analysis of microscopic sections using the DenTiUS Biofilm software

In Experiment 1, 10 to 15 fields or XYZ series were assessed in the central part of the mounted slide for each subject and at each sampling time. In Experiment 2, six fields or XYZ series were evaluated in the central part of the glass disc, again for each subject and at each sampling time. In both experiments, these fields were considered representative of the entire sample after general examination by the observer. The fluorescence emission was determined in a series of XY images, where each image corresponded to a Z position (depth).

Additionally, the optical sections in Experiment 2 were scanned in 0.71 μm portions from the surface of the biofilm to its base, measuring the maximum field thickness (MFT) and the average biofilm thickness of the corresponding sample. The MFT of the biofilm was defined as the distance between the substrate and the peaks of the highest cell clusters.

Data capture was performed using the same settings in all cases, and the spatial scanning mode (XYZ) and a scan format resolution of 2,048 × 2,048 pixels were adopted. The pulsed WLL power was set at 70.00%. The pinhole, zoom, and scanning speed values were 95.50 microns, 1.00%, and 600 Hz, respectively.

The samples were observed using an HCX PL APO CS 63.0 × 1.4 OIL UV objective. A hybrid detector (HyD) was employed to obtain the emission signal from a given specimen for both the green (488 nm) and the red excitation signals (561 nm). The only different sample-dependent values were variations in the laser power of the Acousto-Optic Tunable Filter (AOTF), which ranged between 13.00% and 3.00%. Generally, these parameters were higher in the baseline (i.e., pre-gel) than in the post-gel samples. These values were continuously adjusted to ensure a good quality capture without background noise, avoiding excessive saturation of the brightest pixels in the image. As the microscopy technician was blinded to the experiment, one of the authors was present to instruct that what was seen through the microscope objective be adjusted, thus ensuring that the images were as close as possible to reality.

Cytofluorographic analysis using the Leica confocal software was employed to quantify the BV scores in the XY image series [49]. In this evaluation, the images of each fluorochrome were defined as “channels”, with SYTO™ 9 occupying the green channel and PI the red. The DenTiUS Biofilm software automatically performed the BV calculations [42]. This program utilises the parameters set by the experts, with BV characterised by a high value in the green channel (> 100; range 0 to 255) and a low value in the red (< 100). Bacteria were thus treated as non-viable if the values were > 100 and < 100 in the red and green channels, respectively. High values in both channels (> 100) are visually orange and were also treated as non-viable bacteria.

In more detail, the DenTiUS Biofilm software [42] counts the number of pixels to calculate the BV percentage score for each 0.71 μm section (viable bacteria/viable + non-viable bacteria x 100). Determining the average viability in each field requires sections with a minimum biofilm area and bacterial aggregates of 250 µm^2^ (approximately 4,750 pixels). The program also considers the presence of epithelial cores. These are characterised by compact red areas larger than the bacteria and should not be counted as a non-viable bacterial population. To eliminate these pixels, the software discards epithelial cells, which have a high red channel value, an area bigger than 200 pixels, compact regions with a solidity of more than 0.70, and a minimum mean intensity value of 180. A training set was employed to establish these parameters.

All the results obtained for each section, field, and patient’s discs were stored in a worksheet for subsequent analysis. Before and after removing the epithelial cores, the BV percentage values and their properties were also stored for localisation on the image. The mean BV score for the salivary flora was calculated for the corresponding sample and each biofilm layer in relation to the dental plaque biofilm.

### Statistical analysis

The following five statistical criteria were set for the present study, which has a crossover design: (1) an effect size of 0.25; (2) an alpha error of 0.05; (3) a statistical power of 80%; (4) a number of groups = one; and (5) a number of measures = six. The sample size was calculated using the G*Power 3 program [50], determining that a sample of 20 participants was required.

The statistical analysis was performed using the freely available R software (version 4.4.0) [46]. The quantitative analysis variable in Experiment 1 was the BV in the salivary flora on the overall projection image at the different sampling times. In Experiment 2, these were the bacterial thickness and viability in the overall biofilm structure (overall image projection) and the layers at the different sampling times. The MFT of each field was divided into two equivalent zones: the upper layer and the lower layer. To calculate the thickness of the biofilm layers, it was necessary to obtain the median of each gel at each sample time and determine the minimum value.

The descriptive data for each variable were expressed in terms of the mean and median and their respective measures of dispersion (i.e., standard deviation and interquartile range, respectively). The Shapiro-Wilk test was used to analyse the distribution of the quantitative variables.

As the values of the quantitative variables did not follow a normal distribution, the non-parametric Wilcoxon tests were applied for pairwise comparisons with the corresponding Bonferroni adjustment. Statistical significance was set at *p* < 0.01 (intra-gel analysis) and *p* < 0.008 (inter-gel analysis).

## Results

A total of 56 volunteers were evaluated in our setting, 30 of whom met the predefined criteria to be part of the investigation. One was subsequently excluded from the initial study sample before the start of Experiment 1 due to an unexpected event, meaning that 29 subjects successfully participated in the two experiments using both the test gel and the control gel (Fig. 1). No adverse effects were reported at any stage of the experiments.

### Clinical characteristics of the study group

The mean age of the volunteers was 21.72 ± 3.20 years, with a predominance of females (68.96%). In the whole-mouth assessment, the participants had a mean of 28.55 permanent teeth, and all had very low levels of bacterial plaque (mean = 5.17%). Periodontal parameters indicated that all the volunteers were periodontally healthy (mean BOP = 7.90%; mean PPD = 1.89 mm; mean clinical attachment loss -CAL-= 1.90 mm). All subjects were non-smokers.

### Experiment 1: bacterial viability in saliva

Table 1 contains the BV estimates in saliva for the test gel (CHX + cymenol) and the control gel (CHX) at the different sampling times.

**Table 1:** Bacterial viability in saliva after one application of test and control gels

BACTERIAL VIABILITY IN SALIVA Mean ± Standard Deviation Median (Interquartile Range)										
	BASAL	5 MIN		1 H		3 H		5 H		7 H
**CHX + cymenol**	87.00% ± 6.90% 88.00% (9.00%)	26.50% ± 8.20% 26.30% (26.40%)		28.90% ± 6.50% 27.40% (18.20%)		34.20% ± 16.00% 33.20% (22.80%)		36.90% ± 16.90% 36.40% (21.40%)		40.40% ± 18.60% 40.10% (27.00%)
**CHX**	88.90% ± 8.90% 92.50% (10.60%)	31.60% ± 24.70% 24.50% (33.20%)		36.00% ± 22.10% 36.70% (29.10%)		33.40% ± 14.50% 28.80% (16.90%)		40.10% ± 18.40% 40.00% (26.50%)		37.50% ± 18.10% 37.40% (21.30%)
**INTRA-GEL ANALYSIS** Statistical significance										
	**BASAL**vs.**5 MIN**		**BASAL**vs.**1H** **5 MIN**vs.**1H**		**BASAL**vs.**3H** **5 MIN**vs.**3H**		**BASAL**vs.**5H** **5 MIN**vs.**5H**		**BASAL**vs.**7H** **5 MIN**vs.**7H**	
**CHX + cymenol**	*p* = 7.4506^− 9^		*p* = 7.4506^− 9^ NS		*p* = 7.4506^− 9^ NS		*p* = 7.4506^− 9^ NS		*p* = 7.4506^− 9^ *p* = 0.0044	
**CHX**	*p* = 7.4506^− 9^		*p* = 7.4506^− 8^ NS		*p* = 1.4901^− 8^ NS		*p* = 1.4901^− 8^ NS		*p* = 7.4506^− 9^ NS	
**INTER-GEL ANALYSIS** Statistical significance										
	**BASAL**	**5 MIN**		**1 H**		**3 H**		**5 H**		**7 H**
**CHX + cymenol vs.** **CHX**	NS	NS		NS		NS		NS		NS

CHX: chlorhexidine; H: hours; MIN: minutes; NS: no statistical significance

Wilcoxon test for paired samples. Significance level *p* < 0.01

Wilcoxon test for independent samples. Significance level *p* < 0.008

The mean baseline BV value in the test gel group was significantly reduced five minutes after application (87.00% ± 6.90% *vs.* 26.50% ± 8.20%, *p* = 7.4506^− 9^). A similar performance was demonstrated by the CHX control group (Table 1). In the intra-gel analysis, the significant reduction in BV compared to the baseline values was maintained at all the sampling times for both gels (*p* < 0.01; Table 1). However, a significant recovery of BV was observed in saliva from the five-minute timepoint only for the test gel at seven hours after application (40.40% ± 18.60%, *p* = 0.0044).

On the other hand, in the inter-gel analysis, no significant differences were observed at any sampling time (Table 1).

### Experiment 2: bacterial viability in dental plaque biofilm

The median values of the 48-hour biofilm thicknesses ranged from 11 to 19 μm. Table 2 shows the BV estimates for the total thickness of the dental plaque biofilm achieved by the study gels at different time points. Tables 3 and 4 contain the corresponding values for the upper and lower layers of the biofilm, respectively.

**Table 2:** Bacterial viability in biofilm after one application of the test and control gels

BACTERIAL VIABILITY IN ORAL BIOFILM Mean ± Standard Deviation Median (Interquartile Range)										
	BASAL	5 MIN		1 H		3 H		5 H		7 H
**CHX + cymenol**	91.00% ± 7.60% 93.50% (11.10%)	5.80% ± 14.70% 0.30% (2.90%)		3.70% ± 9.70% 0.20% (1.10%)		7.10% ± 9.30% 2.60% (9.90%)		12.50% ± 16.50% 5.10% (16.90%)		21.30% ± 22.10% 12.50% (38.70%)
**CHX**	88.40% ± 10.30% 91.40% (13.20%)	7.40% ± 15.20% 0.20% (7.50%)		5.30% ± 10.60% 0.40% (2.20%)		11.80% ± 21.10% 1.70% (11.70%)		12.60% ± 17.70% 5.50% (14.00%)		14.30% ± 18.70% 5.50% (14.00%)
**INTRA-GEL ANALYSIS** Statistical significance										
	**BASAL**vs.**5 MIN**		**BASAL**vs.**1H** **5 MIN**vs.**1H**		**BASAL**vs.**3H** **5 MIN**vs.**3H**		**BASAL**vs.**5H** **5 MIN**vs.**5H**		**BASAL**vs.**7H** **5 MIN**vs.**7H**	
**CHX + cymenol**	*p* = 3.7253^− 9^		*p* = 3.7253^− 9^ NS		*p* = 3.7253^− 9^ NS		*p* = 3.7253^− 9^ NS		*p* = 3.7253^− 9^ *p* = 0.0056	
**CHX**	*p* = 3.7253^− 9^		*p* = 3.7253^− 9^ NS		*p* = 3.7253^− 9^ NS		*p* = 3.7253^− 9^ *p* = 0.0002		*p* = 3.7253^− 9^ *p* = 0.0007	
**INTER-GEL ANALYSIS** Statistical significance										
	**BASAL**	**5 MIN**		**1 H**		**3 H**		**5 H**		**7 H**
**CHX + cymenol vs.** **CHX**	NS	NS		NS		NS		NS		NS

CHX: chlorhexidine; H: hours; MIN: minutes; NS: no statistical significance

Wilcoxon test for paired samples. Significance level *p* < 0.01

Wilcoxon test for independent samples. Significance level *p* < 0.008

**Table 3:** Bacterial viability in upper biofilm after one application of the test and control gels

BACTERIAL VIABILITY IN THE UPPER LAYER OF ORAL BIOFILM Mean ± Standard Deviation Median (Interquartile Range)										
	BASAL	5 MIN		1 H		3 H		5 H		7 H
**CHX + cymenol**	91.29% ± 7.79% 93.69% (9.09%)	3.94% ± 10.73% 0.08% (0.12%)		5.42% ± 12.38% 0.29% (1.25%)		8.02% ± 10.14% 2.47% (10.05%)		10.87% ± 16.16% 4.39% (11.92%)		24.13% ± 25.65% 16.34% (37.25%)
**CHX**	89.26% ± 10.21% 92.58% (11.58%)	9.16% ± 19.77% 0.23% (4.55%)		7.44% ± 14.24% 0.76% (5.19%)		14.37% ± 24.60% 1.08% (14.52%)		12.65% ± 18.17% 3.25% (13.77%)		15.96% ± 22.69% 3.48% (20.73%)
**INTRA-GEL ANALYSIS** Statistical significance										
	**BASAL**vs.**5 MIN**		**BASAL**vs.**1H** **5 MIN**vs.**1H**		**BASAL**vs.**3H** **5 MIN**vs.**3H**		**BASAL**vs.**5H** **5 MIN**vs.**5H**		**BASAL**vs.**7H** **5 MIN**vs.**7H**	
**CHX + cymenol**	*p* = 5.9600^− 8^		*p* = 1.4900^− 8^ NS		*p* = 1.1920^− 7^ NS		*p* = 2.9800^− 8^ *p* = 0.0031		*p* = 1.4900^− 8^ *p* = 0.0002	
**CHX**	*p* = 9.5370^− 7^		*p* = 4.7680^− 7^ NS		*p* = 2.3840^− 7^ NS		*p* = 4.7680^− 7^ NS		*p* = 4.7680^− 7^ NS	
**INTER-GEL ANALYSIS** Statistical significance										
	**BASAL**	**5 MIN**		**1 H**		**3 H**		**5 H**		**7 H**
**CHX + cymenol vs.** **CHX**	NS	NS		NS		NS		NS		NS

CHX: chlorhexidine; H: hours; MIN: minutes; NS: no statistical significance

Wilcoxon test for paired samples. Significance level *p* < 0.01

Wilcoxon test for independent samples. Significance level *p* < 0.008

**Table 4:** Bacterial viability in lower biofilm after one application of the test and control gels

BACTERIAL VIABILITY IN THE LOWER LAYER OF ORAL BIOFILM Mean ± Standard Deviation Median (Interquartile Range)										
	BASAL	5 MIN		1 H		3 H		5 H		7 H
**CHX + cymenol**	86.29% ± 12.87% 87.21% (17.71%)	3.83% ± 11.28% 0.09% (0.17%)		9.04% ± 23.06% 0.09% (1.07%)		8.20% ± 10.71% 4.54% (11.96%)		13.08% ± 17.83% 3.88% (18.57%)		22.06% ± 27.65% 9.52% (38.12%)
**CHX**	83.81% ± 18.31% 92.68% (17.96%)	7.82% ± 19.24% 0.29% (1.60%)		8.71% ± 18.24% 0.35% (6.79%)		10.17% ± 18.51% 1.30% (5.99%)		10.58% ± 18.59% 4.63% (9.13%)		10.45% ± 18.62% 1.61% (13.12%)
**INTRA-GEL ANALYSIS** Statistical significance										
	**BASAL**vs.**5 MIN**		**BASAL**vs.**1H** **5 MIN**vs.**1H**		**BASAL**vs.**3H** **5 MIN**vs.**3H**		**BASAL**vs.**5H** **5 MIN**vs.**5H**		**BASAL**vs.**7H** **5 MIN**vs.**7H**	
**CHX + cymenol**	*p* = 1.1920^− 7^		*p* = 2.9800^− 8^ NS		*p* = 1.1920^− 7^ NS		*p* = 2.9800^− 8^ NS		*p* = 7.4510^− 8^ *p* = 0.0065	
**CHX**	*p* = 9.5370^− 7^		*p* = 4.7680^− 7^ NS		*p* = 2.3840^− 7^ NS		*p* = 4.7680^− 7^ NS		*p* = 4.7680^− 7^ NS	
**INTER-GEL ANALYSIS** Statistical significance										
	**BASAL**	**5 MIN**		**1 H**		**3 H**		**5 H**		**7 H**
**CHX + cymenol vs.** **CHX**	NS	NS		NS		NS		NS		NS

CHX: chlorhexidine; H: hours; MIN: minutes; NS: no statistical significance

Wilcoxon test for paired samples. Significance level *p* < 0.01

Wilcoxon test for independent samples. Significance level *p* < 0.008

#### Bacterial viability in the total thickness of dental plaque biofilm

In baseline conditions, the mean BV for the test gel was 91.00% ± 7.60%. After application, it decreased dramatically at five minutes to 5.80% ± 14.70% (*p* = 3.7253^− 9^) and continued to fall at one hour to 3.70% ± 9.70% (*p* = 3.7253^− 9^). These significant reductions in the number of viable microbes compared to the baseline values were also observed for the control gel, as were those obtained at the subsequent sampling times for both products (*p* < 0.01; Table 2). In addition, the restoration of BV from the five-minute estimate was significant for the gel test after seven hours (21.30% ± 22.10%, *p* = 0.0056). Significance in this respect was also observed for the CHX control, in this case, at five and seven hours (Table 2).

Again, no significant inter-gel differences were observed in the total thickness of the dental plaque biofilm at any sampling point (Table 2).

#### Bacterial viability in the layers of dental plaque biofilm

The test gel group had a mean baseline BV of 91.29% ± 7.79% in the upper layer of the dental plaque biofilm and 86.29% ± 12.87% in the lower layer. Five minutes after application, these values dropped dramatically to 3.94% ± 10.73% (*p* = 5.9600^− 8^) and 3.83% ± 11.28% (*p* = 1.1920^− 7^), respectively. A similar performance was found in the control group in both layers (Tables 3 and 4).

As in all previous comparisons, intra-gel analyses revealed a reduction in mean baseline BV values in the upper and lower biofilms across all sampling times for both gels (*p* < 0.01; Tables 3 and 4). Conversely, in the upper layer, only the cymenol-containing gel showed a significant increase at five and seven hours with respect to the five-minute BV values (10.87% ± 16.16%, *p* = 0.0031; 24.13% ± 25.65%, *p* = 0.0002). In the lower biofilm layer, the recovery of the mean BV from the five-minute value was significant only seven hours after application of the test gel (22.06% ± 27.65%, *p* = 0.0065).

Once more, no significant inter-gel differences were observed in the upper and lower biofilm layers at any sampling point (Tables 3 and 4).

## Discussion

### Methodological approach

Historically, different microscopic techniques have been used to visualise the salivary and oral biofilm microbial communities, including epifluorescence, transmission, and scanning electron microscopy [51–53]. These methods can, however, produce blurred or distorted images, making accurate analyses difficult, especially in fluid-filled structures [54, 55]. The use of CLSM overcomes, or at least mitigates, this disadvantage and is noteworthy for its resolution, optical-sectioning capability (0.5–2.0 μm), and versatility with 3D imaging [55]. Additionally, it facilitates real-time observations and enables analyses of dental plaque biofilm samples to be performed without altering their delicate structure [56].

Furthermore, even after microscopy images were obtained, the earliest studies had to quantify the number of microorganisms manually or semi-automatically, which is a slow, laborious, and error-prone process [38]. Nowadays, using software to conduct bacterial counts automatically ensures that quick, accurate, reliable, and repeatable results can be obtained [39–41]. This is very important when comparing outcomes and reaching solid conclusions. To this end, our research team has developed the DenTiUS Biofilm software [42], which is a toolbox designed to determine the BV of individual samples automatically and remove epithelial cell nuclei from the calculation.

Several CLSM investigations have evaluated the in situ BV of CHX, either alone [31, 34] or in comparisons with EOs [28, 33, 37], using mouthwash applications and oral biofilm samples to do so. For EOs alone, the in situ substantivity of mouthwashes with *vs.* without alcohol has also been analysed in the oral biofilm [32]. Concerning cymenol specifically, only two studies to date have assessed its antimicrobial activity in in vitro [29] or in situ [43] experiments, reflecting how little investigation has been done on this compound. The sole in vivo study compared cymenol *vs.* CPC rinses in saliva, while the only in vitro research used a multispecies biofilm model to evaluate the same gel formulations as in the current work. There are, however, no in situ studies available about the latter despite the fact that substantivity is a property of antiseptics that can only be assessed in living humans and cannot be determined in artificial environments [43].

To the best of our knowledge, this is the first in situ clinical trial to use CLSM and the DenTiUS Biofilm software to analyse the antimicrobial effect and in situ substantivity of a new 0.20% CHX + cymenol gel *vs.* the traditional 0.20% CHX gel formulation. Specifically, saliva and dental plaque biofilm samples were collected from 29 healthy volunteers up to a maximum of seven hours after applying each of the two gels.

**Fig. 3:**
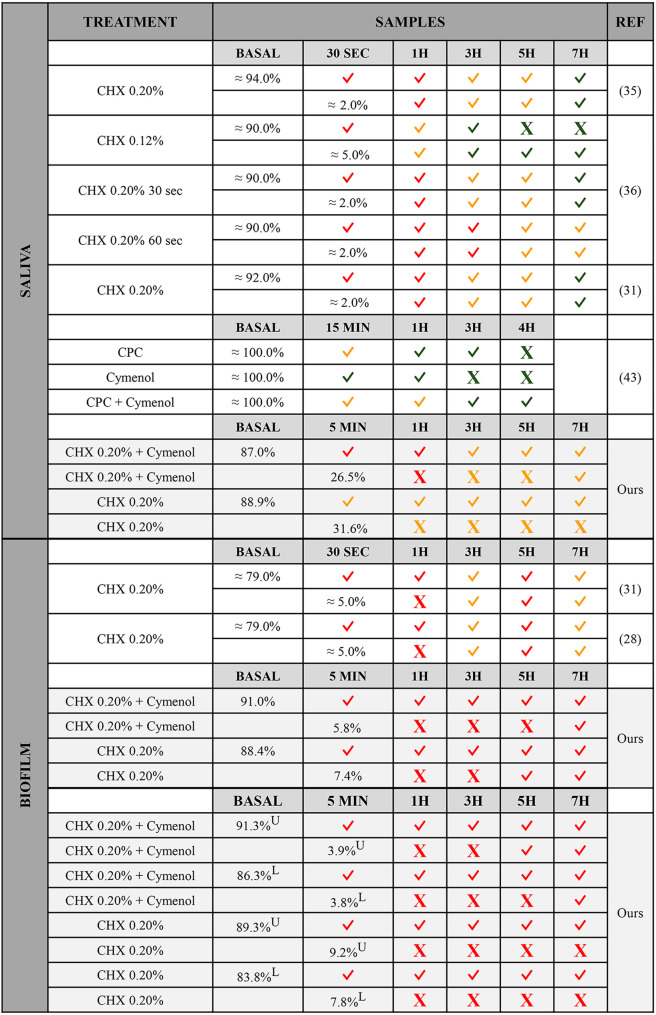
Bacterial viabilities in confocal microscopy studies assessing chlorhexidine and/or cymenol in saliva and biofilm The table summarises the main findings obtained in the present research (gel) and previous articles (mouthwashes) using confocal laser scanning microscopy to study the antimicrobial activity and substantivity of chlorhexidine and/or cymenol in saliva or biofilm. Intra-group comparisons of baseline *vs.* the different sampling moments, and immediate *vs.* the rest of the moments are reflected. Obtaining statistical significance in a given comparison is indicated by the symbol “✓”, while “X” indicates the absence of differences. The colour of the symbols for each treatment at each sampling time point indicates the range of bacterial viability so that green corresponds to ≤ 100.00%-≥60.00%, orange to < 60.00%-≥30.00%, and red to < 30.00%-≥0.00% CHX: chlorhexidine; CPC: cetylpyridinium chloride; H: hour; L: lower layer; MIN: minute; REF: references; SEC: second; U: upper layer

In Fig. 3, we summarise the main findings of our research *vs.* those described in CLSM studies to date on the antimicrobial activity and substantivity of CHX and/or cymenol in saliva and biofilm applied by rinse or gel. Given that most of the found investigations belong to our research team [28, 31, 35, 36], we can affirm that the methodology and statistical analyses performed are similar, making the results more comparable.

### Bacterial viability in saliva

When applied via mouthwash, a single dose of CHX at concentrations of 0.12-0.20% has demonstrated lower BV values as an immediate effect (30 s = 0.00–4.00%) [31, 35, 36] than those obtained here with a CHX 0.20% gel application (five minutes = ~ 32.00%). Similar observations have been reported one hour after rinsing with CHX at 0.20% for 30 or 60 s (9.00–10.00% [31, 35, 36] *vs.* 36.00%), and even three hours after rinsing for 60 s (16.00% [36] *vs.* ~ 33.00%). However, three hours after usage, the number of viable bacteria identified in our CHX 0.20% gel was lower, or at least similar, to that observed with CHX 0.12% or 0.20% rinses (33.00% *vs.* 72.00% [36] and 30.00% [31, 35, 36]). What is more striking is the fact that the gel seems to maintain the antibacterial effect better over several hours than is achieved with rinsing (gel at five hours = ~ 40.00%, and seven hours = ~ 38.00%; mouthwash at five hours = 85.00–55.00%, and seven hours = 90.00–58.00%) [31, 34–36].

When it comes to the antimicrobial activity of cymenol in saliva, this is, as expected, more potent when combined with CHX rather than with CPC, as in the current research [43]. This is true for both the immediate effect (CHX + cymenol at five minutes = ~ 27.00% *vs.* CPC + cymenol at 15 min = ~ 47.00%) and the short-term effect (one hour = ~ 29.00% *vs.* ~ 59.00%; three hours = ~ 34.00% *vs.* ~ 67.00%; five hours = ~ 37.00% *vs.* four hours = ~ 81.00%) [43]. Conversely, although the addition of cymenol to a CPC mouthwash prolongs the substantivity of the latter by more than an hour [43], we found no significant differences when comparing the BV values of CHX with *vs.* without cymenol over our time points.

### Bacterial viability in dental plaque biofilm

In the literature, authors have commonly evaluated BV by dividing the entire biofilm into three layers: outer [layer 1], middle [2], and inner [3] [28, 31–34]. In terms of the immediate effect, our test gel had microbial viabilities in the upper and lower layers of around ~ 4.00%, which is similar to those described 30 s after rinsing with 0.20% CHX (layers 1 and 3 = ~ 5.00%) [31, 33]. Our control group demonstrated slightly higher values (~ 9.00% and ~ 8.00%), closer to those of EO mouthwashes (layers 1 and 3 = ~ 7.00%) [32]. Seven hours after usage, the gels showed levels of microbial viability in both the upper and lower layers of ~ 24.00% and ~ 22.00% for the CHX + cymenol and ~ 16.00% and ~ 11.00% for the CHX control. These estimates are lower than those described in superficial and deep layers for rinses of 0.20% CHX after up to eight hours (range: layer 1 = 27.00%-~40.00%; range: layer 3 = ~ 36.00%-~40.00%) [28, 31, 34], or for EOs in superficial zones after seven hours (range: ~35.00%-~42.00%) [28, 32].

In contrast to the results of a recent in vitro trial, in which higher bactericidal activity and penetrability into the biofilm were observed for CHX + cymenol gel than for that with CHX-only [29], we observed no differences between the mean BVs of these gels at any sampling time or for any given layer.

### Bacterial viability in saliva and dental plaque biofilm: explanation of the differences with other studies

In the present study, the effect of the two gels tested in saliva and dental plaque biofilm was maintained up to seven hours after application (i.e., the difference at baseline *vs.* seven hours was significant). Moreover, in light of the discussed above when comparing our findings to those obtained in previous research, the immediate antimicrobial effect of 0.20% CHX on both niches is greater when applied by rinse than by gel, whether alone or with cymenol. On the contrary, the CHX effect is maintained longer when applied by gel, alone or with cymenol, than if used alone via mouthwash.

The mechanical action involved in the rinsing process may favour a better antiseptic distribution in the oral environment, implying a greater immediate effect on the flora. This is in line with the finding that an in vivo active mouthwash of CHX or EOs is more effective than the ex vivo passive immersion in these agents [37]. As previously described [22], the better maintenance of the antimicrobial effect of the gel formulations over time can be attributed to their mucoadhesive properties. Adhesion is one of the multiple factors that determine the substantivity of an antiseptic [43] and may explain the best results for gels *vs.* mouthwashes. The product can remain in the oral cavity longer, and the active ingredient can be retained and released for extended periods [22]. Furthermore, preference for gel over rinse implies other advantages, such as the usage in individuals who cannot effectively control the swallowing reflex (i.e., children or people with disabilities), preventing the risk of ingestion [22].

On the other hand, unlike previous in vivo research on 0.05% CPC mouthwashes in saliva [43] and in vitro experiments with 0.20% CHX gel in biofilm [29], the addition of cymenol to a 0.20% CHX gel does not enhance the already potent in vivo antimicrobial effect of the gold-standard antiseptic. Regarding the first observation, CPC products at concentrations above, equal to, or below 0.05% are not among the best for plaque control [57]. CHX at 0.20% has shown an optimal inhibition of plaque formation, greater than when applied at 0.12% or 0.06% [58, 59]. Adding cymenol to 0.05% CPC might enhance the mechanism of action of the latter, addressing its weaknesses and improving its performance. In contrast, adding such EO to 0.20% CHX does not improve the activity of the gold-standard antiseptic, which might already be operating close to its maximum potential. Concerning the in vitro - in vivo differences, host-related factors, including immunity and host/microbe interaction, micro-environmental components, or factors related to the contact time and diffusion of the antimicrobial may influence the discrepancy in the results obtained [60, 61].

## Conclusions

A single application of a gel containing CHX + cymenol demonstrates potent and immediate antimicrobial activity on salivary flora and the *de novo* dental plaque biofilm (both at the upper and lower levels). Its substantivity in both oral niches persists seven hours after a single application. However, similar effects are obtained with a gel containing CHX alone.

## Electronic supplementary material

Below is the link to the electronic supplementary material.

**Additional file 1:** Protocol of the present study.

**Additional file 2:** CONSORT checklist.

## Data Availability

Data will be made available on a case-by-case basis, and additional information can be obtained by contacting the corresponding author.
