# Supplementary material for: Randomised-crossover clinical trial on the substantivity of a single application of a gel containing chlorhexidine and o-cymen-5-ol on the oral biofilm and saliva
Source: BMC Oral Health. 2024 Oct 19;24:1247. doi: 10.1186/s12903-024-05042-7 (PMC11490038; doi:10.1186/s12903-024-05042-7)
Supplement: Supplementary file 1 — Additional file 1: Protocol of the present study. [file 12903_2024_5042_MOESM1_ESM.docx]

Additional file 1. Protocol of the present study.

**MATERIAL AND METHODS**

**1-Legal considerations to be taken into account for applied oral antiseptics**

The development of this clinical research requires the authorisation of the Autonomic Ethics Committee of Galicia (registration code 2021/478).

**2-Definition of the study design**

The project will be a clinical investigation with a crossover, parallel, triple-blind, randomised, balanced allocation design about the utilised gels. The study design will meet, where applicable, the requirements of the CONSORT checklist, and the study protocol will be published on ClinicalTrials.gov (<https://clinicaltrials.gov/>).

**3-Setting of the study, recruitment of participants, inclusion and exclusion criteria, withdrawal criteria**

The project will take place at the Faculty of Medicine and Dentistry of Santiago de Compostela (University of Santiago de Compostela, Spain), from where the voluntary enrolment of the participants will be sought between September 2022 and June 2023. Two dentists will evaluate all the volunteers dentally and periodontally. For their dental status, the clinical presence of untreated carious lesions will be determined; for their periodontal status, the bacterial plaque level (BPL) and bleeding on probing (BOP) in the entire mouth (six locations per tooth) will be evaluated using a binary scale (presence/absence). The probing pocket depth (PPD) and clinical attachment level (CAL) in the full mouth (six locations per tooth) will also be assessed in each patient using a PCP-UNC 15 probe.

The inclusion criteria will be as follows: a) systemically healthy adult volunteers aged 20-45 years with an excellent oral health status, b) a minimum of 24 permanent teeth with no evidence of gingivitis or periodontitis (BOP <10%, PPD ≤3 millimetres -mm-) (1) and c) no untreated caries at the start of the study. The following exclusion criteria will be applied: a) smoker or ex-smoker, b) the presence of dentures or orthodontic appliances, c) allergies to oral hygiene products, d) antibiotic treatment or the routine use of oral antiseptics in the previous three months and e) the presence of any systematic disease that alters the production or composition of saliva. Written informed consent will be obtained from all the volunteers.

**4-Sample size calculation, study group, and gels applied, randomisation procedure**

Since this project will involve a crossover design, we have determined an effect size of 0.25, an alpha error of 0.05 and a power of 0.80, for which a minimum sample of 20 participants would be required. Due to the possible loss of patients for various reasons, the initial study group will consist of 30 participants. The sample size was calculated with the statistical software G*Power 3 program (2).

The sample of 30 participants will participate in two experiments, each requiring two appointments (Additional Figure 1):

1) Experiment 1 – Salivary flora: Unstimulated saliva samples (1 millilitre -ml-) will be collected via the spitting method (3) from each volunteer at baseline, five minutes, one, three, five, and seven hours after applying the gel. The researcher will apply the corresponding gel on the buccal and palatal/lingual tooth surfaces and gingival mucosa of both arches of the subject´s mouth.

The gels that will be used are as follows:

- Test gel: a 0.20% Lacer® CHX gel containing a new cymenol formulation.

- Control gel: a conventional 0.20% Lacer® CHX gel without cymenol.

2) Experiment 2 – Dental plaque biofilm: An intraoral overlapping disc-holder splint device, which consists of two splints, each of which contains six two-mm-diameter circular cavities (IDODS, registered patent number: ES 2380252 B2), will be created for each volunteer. The volunteers will wear the IDODS for 48 hours to encourage dental plaque biofilm growth on the glass discs. They will be instructed only to remove it during meals. After 48 hours, the glass discs will be removed one by one at the timepoints described above. The investigator will apply the corresponding gel to the entire exposed surface of the six discs *ex vivo* to ensure the integrity of the biofilm. After this application, the volunteers will place the IDODS back inside their mouths until the final sampling time of seven hours.

For both experiments, they will be told not to brush their teeth or practice other oral hygiene measures between 48 hours before the sampling day and when the last specimen will be taken. At the sampling appointments, they will not be allowed to eat or drink from 60 minutes before the collection of the first oral sample until after the last one is obtained. This process will start at 8.45 a.m. (baseline sample) and end at 4.00 p.m. (sample obtained seven hours after gel application). Both types of gels (test and control) will be presented in opaque tubes labelled with a participant number (from one to 30), and each one will contain the amount required for a single application plus extra gel in the case of possible losses.

A balanced randomisation process will be adopted for the designation list using the freely distributed R software (4), indicating which gel each participant will receive during each experiment's first and second appointments. The list of designations will be available in an Excel file. All the volunteers will have the two study gels applied in both experiments, with a minimum period of two weeks between them.


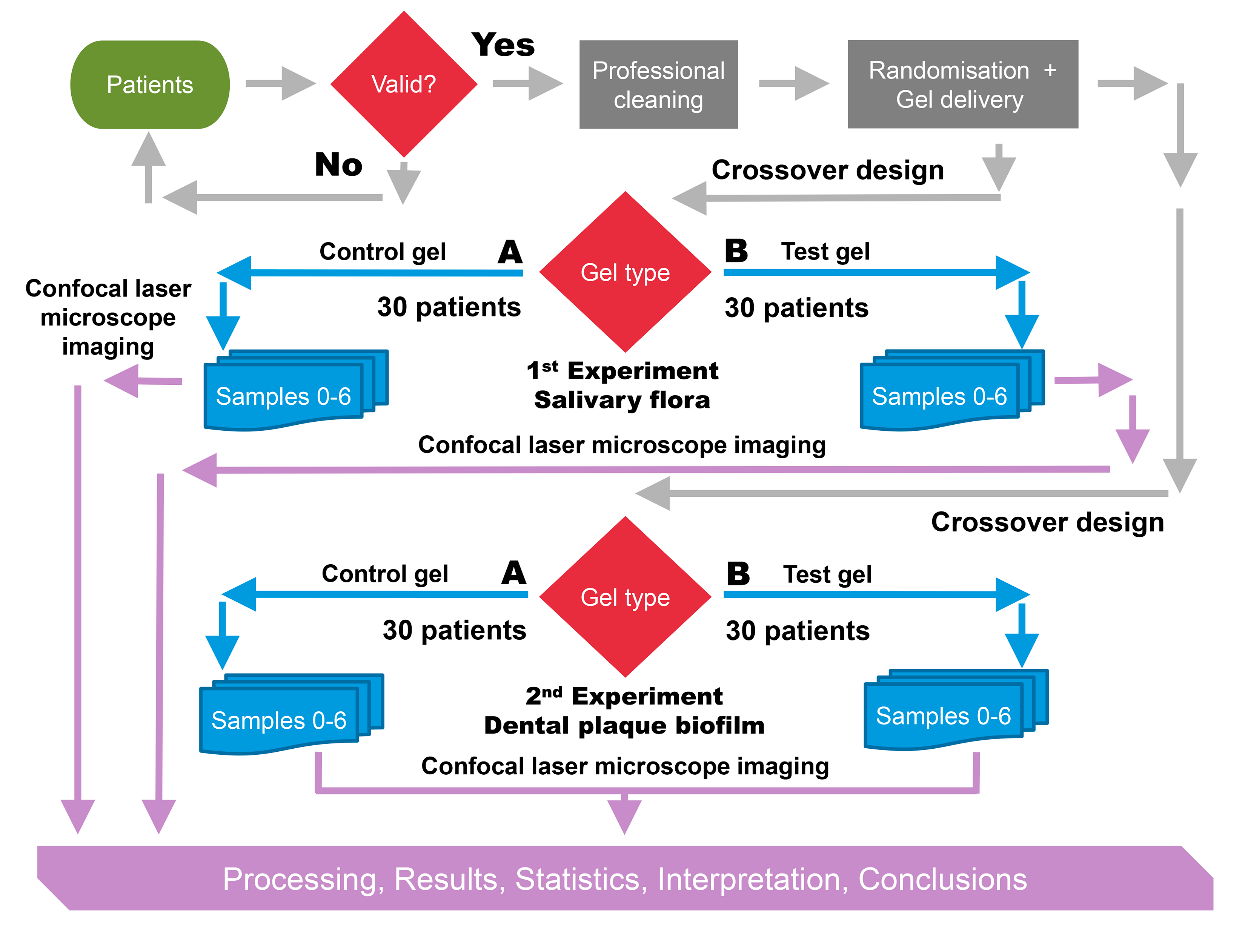


**Additional figure 1.** The research protocol applied in this project.

**5- Processing of the saliva and dental plaque biofilm samples using confocal laser microscopy**

The LIVE/DEAD® BacLight™ kit (Molecular Probes, Leiden, the Netherlands), consisting of SYTO™ 9 and propidium iodide (PI), will be used as a fluorescence solution to determine bacterial viability (BV) in the two experiments. The solution will be prepared according to the manufacturer's instructions, i.e., using 5 ml of sterile water, filtered with a 0.22 micrometer (µm) Millipore membrane filter (Millipore Ibérica S.A., Madrid, Spain) to achieve a 1:1 ratio of both fluorochromes, and stored at -20°C. As bacteria with intact or damaged cytoplasmic membranes emit green and red fluorescence, respectively, the dual staining will differentiate them (5).

In Experiment 1, the saliva samples will be centrifuged at 14,500 rpm for 10 minutes. The supernatant will be discarded, and the pellet obtained will be re-suspended in 100 microliters (µl) of sterile water with 100 µl of the fluorescence solution. The bacterial suspension will be shaken for homogenisation and stored in the dark at room temperature for 15 minutes.

In Experiment 2, the glass discs removed from the IDODS will be immediately immersed in 150 µl of the LIVE/DEAD® BacLight™ fluorescent solution and also kept in a dark chamber at room temperature for 15 minutes.

The microscopic observations in both experiments will be carried out by a researcher unaware of the study design. To this end, a Leica TCS SP5 X laser scanning spectral confocal microscope (Leica Microsystems Heidelberg GmbH, Mannheim, Germany) equipped with a white laser (WLL) (Research Infrastructures Area of University of Santiago de Compostela. Microscopy Unit, CIMUS) will be employed.

**6-Analysis of microscopic sections using the DenTiUS Biofilm software**

In Experiment 1, 10 to 15 fields or XYZ series will be assessed in the central part of the mounted slide for each subject and at each sampling time. In Experiment 2, six fields or XYZ series will be evaluated in the central part of the glass disc, again for each subject and at each sampling time. In both experiments, these fields will be considered representative of the entire sample after general examination by the observer. The fluorescence emission will be determined in a series of XY images, where each image corresponds to a Z position (depth).

Additionally, the optical sections in Experiment 2 will be scanned in 0.71 µm portions from the surface of the biofilm to its base, measuring the maximum field thickness (MFT) and the average biofilm thickness of the corresponding sample. The MFT of the biofilm will be defined as the distance between the substrate and the peaks of the highest cell clusters.

Data capture will be performed using the same settings in all cases. The spatial scanning mode (XYZ) and a scan format resolution of 2,048 x 2,048 pixels will be adopted. The pulsed white laser (WLL) power will be set at 70.00%. The pinhole, zoom, and scanning speed values will be 95.50 microns, 1.00%, and 600 Hz, respectively.

The samples will be observed using an HCX PL APO CS 63.0 x1.4 OIL UV objective. A hybrid detector (HyD) will be employed to obtain the emission signal from a given specimen for both the green (488) and the red excitation signals (561). The only different sample-dependent values will be variations in the laser power of the Acousto-Optic Tunable Filter (AOTF). Generally, these parameters will be higher in the baseline (i.e., pre-gel) than in the post-gel samples. These values will always be adjusted to ensure a good quality capture without background noise, avoiding excessive saturation of the brightest pixels in the image. As the microscopy technician will be blinded to the experiment, she will be instructed to constantly adjust what will be seen through the microscope objective, thus ensuring that the images will be as close as possible to reality.

Cytofluorographic analysis using the Leica confocal software will be employed to quantify the BV scores in the XY image series (6). In this evaluation, the images of each fluorochrome will be defined as "channels", with SYTO™ 9 occupying the green channel and PI the red. The DenTiUS Biofilm software will be used to perform the BV calculations automatically (7). This program utilises the parameters set by the experts, with BV characterised by a high value in the green channel (>100; range 0 to 255) and a low value in the red (<100). Bacteria will be thus treated as non-viable if the values will be >100 and <100 in the red and green channels, respectively. High values in both channels (>100) are visually orange and will also be treated as non-viable bacteria.

In more detail, the DenTiUS Biofilm software (7) counts the number of pixels to calculate the BV percentage score for each 0.71 µm section (viable bacteria/viable + non-viable bacteria x 100). Determining the average viability in each field requires sections with a minimum biofilm area and bacterial aggregates of 250 µm^2^ (approximately 4,750 pixels). The program also considers the presence of epithelial cores. These are characterised by compact red areas larger than the bacteria and should not be counted as a non-viable bacterial population. To eliminate these pixels, the software discards epithelial cells, which have a high red channel value, an area bigger than 200 pixels, compact regions with a solidity of more than 0.70 and a minimum mean intensity value of 180. A training set will be employed to establish these parameters.

All the results obtained for each section, field, and patient’s discs will be stored in a worksheet for subsequent analysis. Before and after removing the epithelial cores, the BV percentage values and their properties will also be stored for localisation on the image. The mean BV score for the salivary flora will be calculated for the corresponding sample and each biofilm layer in relation to the dental plaque biofilm.

**7-Statistical analysis**

The statistical analysis will be performed using the freely available R software (version 4.4.0) (4). The quantitative analysis variable in Experiment 1 will be the BV in the salivary flora on the overall projection image at the different sampling times. In Experiment 2, these will be the bacterial thickness and viability in the overall biofilm structure (overall image projection) and the layers at the different sampling times. The MFT of each field will be divided into two equivalent zones: the upper layer and the lower layer. To calculate the thickness of the biofilm layers, it will be necessary to obtain the median of each gel at each sample time and determine the minimum value.

The descriptive data for each variable will be expressed in terms of the mean and median and their respective measures of dispersion (i.e., standard deviation and interquartile range, respectively). The Shapiro-Wilk test or the Kolmogorov-Smirnov test will be used to analyse the distribution of quantitative variables.

Assuming normal distribution of the values of the quantitative variables, the one-factor repeated measures ANOVA test will be applied for intra-gel comparisons (same gel) between different times of collection of saliva samples and dental plaque biofilm, and inter-gel comparisons (between different gels) for the same time of collection of saliva samples and dental plaque biofilm. If the values of the quantitative variables do not have a normal distribution, the non-parametric Wilcoxon tests for paired (intra-gel analysis) and independent (inter-gel analysis) samples will be applied for pairwise comparisons with the corresponding Bonferroni adjustment. Since this project will involve a crossover design, we have determined an effect size of 0.25, an alpha error of 0.05 and a power of 0.80 for which a minimum sample of 20 participants would be required. Statistical significance will be set at p <0.01 (intra-gel) and p<0.008 (inter-gel).

**REFERENCES**

(1) Papapanou PN, Sanz M, Buduneli N, Dietrich T, Feres M, Fine DH, et al. Periodontitis: consensus report of workgroup 2 of the 2017 world workshop on the classification of periodontal and peri-implant diseases and conditions. J Periodontol. 2018; 89 Suppl 1:S173-S182.

(2) Faul F, Erdfelder E, Lang A, Buchner A. G*Power 3: a flexible statistical power analysis program for the social, behavioral, and biomedical sciences. Behav Res Methods. 2007; 39(2):175-191.

(3) Navazesh M, Christensen CM. A comparison of whole mouth resting and stimulated salivary measurement procedures. J Dent Res. 2014; 61(10):1158.

(4) R Core Team. R: a language and environment for statistical computing. Vienna, Austria: R Foundation for Statistical Computing; 2024. Available at: <https://www.R-project.org/>.

(5) Thermo Fisher Scientific. LIVE/DEAD™ BacLight™ Bacterial Viability Kits [Internet]. Waltham (MA), United States of America: Thermo Fisher Scientific Inc.; 2024. Available at: <https://www.thermofisher.com/order/catalog/product/L7007?SID=srch-srp-L7007>.

(6) Leica Microsystems. Leica Application Suite X (RRID:SCR_013673). Wetzlar, Germany; 2023. Available at: <https://www.leica-microsystems.com/products/microscope-software/details/product/leica-las-x-ls/>.

(7) Vila-Blanco N, Tomás I, Balsa-Castro C, Carreira MJ. DenTiUS Biofilm software. Santiago de Compostela, Spain: CiTIUS, USC; 2017. Available at: <https://gitlab.citius.gal/DenTiUS/DentiusBiofilm>.
